# tRF-1:28-Val-CAC-2 promotes the development of nasopharyngeal cancer by targeting EPHB2

**DOI:** 10.3389/fonc.2025.1564601

**Published:** 2025-05-23

**Authors:** Hui Li, Xiaomin Wang, Anchi Sun, Weiwei Liu, Rongrong Lv, Mingjie Zhang, Zhiwei Xing, Shiyin Ma, Yehai Liu, Kai Zhang

**Affiliations:** ^1^ Department of Otolaryngology-Head and Neck Surgery, The First Affiliated Hospital of Anhui Medical University, Hefei, Anhui, China; ^2^ Department of Otolaryngology-Head and Neck Surgery, The First Affiliated Hospital of Bengbu Medical University, Bengbu, Anhui, China; ^3^ Anhui Engineering Technology Research Center of Biochemical Pharmaceutical, Bengbu, Anhui, China; ^4^ Department of Stomatology, The First Affiliated Hospital of Bengbu Medical University, Bengbu, Anhui, China

**Keywords:** tRF-1:28-Val-CAC-2, *EPHB2*, NPC, tsRNA, EMT

## Abstract

**Introduction:**

Nasopharyngeal carcinoma (NPC) is highly aggressive, with a particularly high incidence in South China. is an aggressive cancer that affects particularly high numbers of patients in southern China and Southeast Asia. The cure rate of previous treatments is decreasing year by year, underscoring the need to devise new approaches to treating affected patients. This study was developed to examine the tRF-1:28-Val-CAC-2 expression in NPC and to elucidate its effects on proliferative, migratory, and apoptotic dynamics in NPC cells.

**Methods:**

RT- qPCR was used to quantify tRF-1:28-Val-CAC-2 expression in NPC cells. Transfection was used to manipulate tRF-1:28-Val-CAC-2 expression levels, and proliferation, migration, and invasion were then evaluated through CCK-8, wound-healing, colony formation, and Transwell approaches. Apoptotic induction and cell cycle progression were assessed through flow cytometry, while EMT-related marker expression was assessed via qPCR and Western immunoblotting. The effects of tRF-1:28-Val-CAC-2 on the growth and distant metastasis of tumors were then tested *in vivo* using nude mice.

**Results:**

NPC cells exhibited tRF-1:28-Val-CAC-2 upregulation that was associated with significantly increased proliferative, migratory, and invasive activity together with the suppression of apoptotic death. *In vivo* experiments further confirmed the ability of tRF-1:28-Val-CAC-2 to promote tumor growth and distant metastasis. At a mechanistic level, these effects were related to the control of *EPHB2* gene expression by tRF-1:28 Val-CAC-2, thereby shaping the survival and malignancy of the cells.

**Discussion:**

These results demonstrate that tRF-1:28-Val-CAC-2 promoted *EPHB2* to enhance tumorigenic behavior in NPC cells, underscoring its key role as a novel target for therapeutic intervention.

## Graphical Abstract

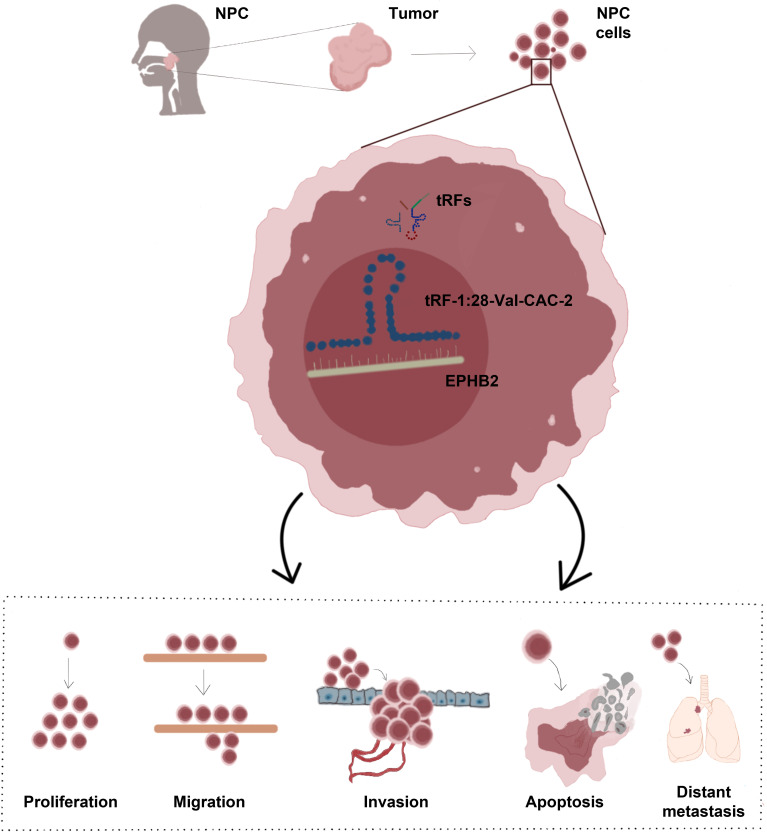

## 1 Introduction

NPC is prevalent in southern China, North Africa, and Southeast Asia, with high incidence rates being attributable to genetic risk factors, Epstein-Barr virus infection, and regional dietary habits (1). During the early stages of disease progression, NPC tumors generally respond to radiotherapeutic and chemotherapeutic treatment, with adjuvant chemoradiotherapy often affording improved survival rates to patients. More advanced NPC, however, is prone to chemoresistance, metastatic progression, and recurrence, and it thus remains a persistent clinical challenge (2). There is thus a pressing need to design new approaches to NPC management.

The emergence of more advanced sequencing technologies over the past decade has fueled the identification of a growing variety of non-coding RNAs present within the human genome. These include transfer RNA-derived small RNAs (tsRNAs), which are obtained from mature tRNAs or pre-tRNAs and are classified as tRNA halves and tRNA-derived fragments (tRFs), the latter of which are additionally subcategorized into endogenous tRFs, and tRF-1/2/3/5 transcripts (3). There have been past reports linking certain types of tsRNAs to cancer, with tRF-30-JZOYJE22RR33 and tRF-27-ZDXPHO53KSN, for instance, having been upregulated to a significant degree in trastuzumab-resistant breast cancer such that they have been proposed to influence drug resistance (4). Mao et al. detected higher levels of tRF-17-18VBY9M expression in the serum and tumor tissues of gastric cancer patients (5), while in NSCLC, a reduction in the levels of i-tRF-AspGTC and tRF-1-SerCGA has been reported in serum exosomes (6). Zhang et al. additionally noted that tRF-33 offers prognostic and diagnostic utility in gastric cancer, targeting the STAT3 pathway to suppress the viability of tumor cells (7). There are also many reports in which tsRNAs have been advanced as promising therapeutic targets in cancers and other disease types (8–11).

The epithelial-mesenchymal transition (EMT) is a key process in tumorigenesis whereby epitheloid cells go through mesenchymal-like changes conducive to their migratory and invasive growth, ultimately facilitating tumor progression. Non-coding RNAs have recently been found to interact with EMT pathways to facilitate malignant tumor progression. For instance, miR-875-5p can suppress EMT induction to constrain the growth and metastasis of cervical cancer cells (12), while LINC00930 overexpression in pancreatic cancer inhibits the EMT (13). CircRREB1 found to promote pancreatic ductal adenocarcinoma progression (14).

The Eph receptor family tyrosine kinase EPHB2 has an expression profile that is often dysregulated in various types of disease (15–17), with its expression often being positively correlated with tumor metastasis (18). EPHB2 has been linked to NPC recurrence, and its high expression levels are predictive of poor NPC patient outcomes (19).

In preliminary analyses, our team identified tRF-1:28-Val-CAC-2 as a novel tsRNA that was significantly upregulated in NPC tumors and promote the growth of tumor cells (20). As bioinformatics analyses suggested that EPHB2 may be a downstream target of this tsRNA, this study was developed to explore how changing tRF-1:28-Val-CAC-2 expression within NPC cells modulates their proliferative and EMT activity. To complement these experiments, animal model experiments were conducted aimed at validating the role of this tsRNA in NPC development and to clarify the degree to which it can modulate tumor growth through the regulation of EPHB2 expression.

## 2 Methods

### 2.1 Cells and treatments

– Nasopharyngeal epithelial cell NP69、EBV-positive NPC cell C666–1 and EBV-negative NPC cells S26、HNE1、CNE2Z were acquired from School of Pharmacy, Bengbu Medical University (Anhui, China), stored at -80°C, thawed, and cultured with DMEM or RPMI-1640 containing 10% FBS and penicillin/streptomycin in a CO_2_ incubator.

Lipofectamine 2000 (Invitrogen, USA) was used as directed for transfection with tRF-1:28-Val-CAC-2 mimic, inhibitor, or negative control (NC or anti-NC) constructs. Experiments were performed after 48 h.

### 2.2 Real-time quantitative polymerasechain reaction

The AG RNAex Pro RNA Extraction Reagent (AG21102, Accurate Biotechnology) was used to extract cellular RNA, after which Evo M-MLV Reverse Transcriptase (AG11705, Accurate Biotechnology) was used for cDNA preparation. A SYBR Green Pro Taq HS Kit (AG11702, Accurate Biotechnology) was used to assess gene expression, using GAPDH or U6 as reference controls. The 2^−ΔΔCt^ method was applied for determining gene expression. Utilized primers are presented in **Table 1**.

**Table 1:** qPCR primers and transfected constructs.

Gene	Primer Sequences(5’to3’)
Human GAPDH	GGAGCGAGATCCCTCCAAAAT
	GGCTGTTGTCATACTTCTCATGG
E-cadherin	CGAGAGCTACACGTTCACGG
	GGGTGTCGAGGGAAAAATAGG
Fibronectin	CGGTGGCTGTCAGTCAAAG
	AAACCTCGGCTTCCTCCATAA
Vimentin	GACGCCATCAACACCGAGTT
	CTTTGTCGTTGGTTAGCTGGT
tRF-1:28-Val-CAC-2 mimics antisense	AACGUGAUAACCACUACACUACAGAAGC
tRF-1:28-Val-CAC-2 mimics sense	GCTTCTGTAGTGTAGTGGTTATCACGTT
tRF-1:28-Val-CAC-2 inhibitor	AACGUGAUAACCACUACACUACAGAAGC
tRF-1:28-Val-CAC-2 NC	UUCUCCGAACGUGUCACGUdTdT
	ACGUGACACGUUCGGAGAAdTdT

### 2.3 Proliferation assessments

Proliferation was examined using CCK-8 assays (B1099; BIOMEOICAL). Cells plated in 06-well plates (5,000/well) were cultured for 1–6 days, after which CCK-8 reagent (10 μl/well) was introduced for 2 h at 37°C followed by the reading of absorbances (450 nm) with a microplate reader (Infinite F50; Tecan). Analyses were performed in triplicate.

Colony formation assays were conducted by seeding cells in 6-well plates (600/well) and culturing them for 14 days, after which they were fixed (4% paraformaldehyde, 30 min), stained for 30 min using crystal violet (Sigma, C0775), rinsed using ddH_2_O, and imaged via microscopy (MF53; Mshot). ImageJ (NIH, MD, USA) was used for image analysis, and assays were conducted in triplicate.

### 2.4 Migration assays

For wound healing assays, cells were cultured in 6-well plates until 90% confluent. Scratches were made in the monolayer with a 10 μl pipette tip. Imaging was then performed via microscopy after 0, 16, and 24 h, and the resultant images were used to compute the rate of cellular migration.

Transwell migration assays were performed with Transwell chambers (Corning). After adding 2×10^4^ cells in medium with no serum to the upper portion of these chambers, serum-containing medium was put in the lower chamber, and kept for 48 h at 37°C. Then, any cells remaining on the upper surface were discarded, while 4% paraformaldehyde was used to fix cells that had migrated into the lower chamber, followed by staining with crystal violet, imaging, and counting via microscopy (100× magnification, 3 fields/well).

### 2.5 Invasion assays

Transwell chambers precoated with Matrigel were used; otherwise, all assay protocols were identical to those for the migration assay.

### 2.6 Flow cytometry

Apoptosis was analyzed by harvesting cells, rinsing, resuspending them in PBS, and staining as directed with annexin V-FITC and PI (Beyotime, C1062S). Levels of apoptosis were then assessed by analyzing cells with a flow cytometer (NovoCyte, 2060R).

Cell cycle analyses were performed by fixing cells overnight at 4°C in 70% ethanol, rinsing them using PBS, and staining them using PI before analysis by flow cytometry to evaluate cell cycle distributions.

### 2.7 Western immunoblotting

RIPA buffer with PMSF was used for protein extraction, with concentrations then being monitored using the BCA method. Proteins (30 μg) were run SDS-PAGE and electroblotted to PVDF membranes, which were blocked (5% skimmed milk/TBST, room temperature, 2 h), probed overnight with primary antibodies at 4°C, rinsed with TBST, treated with secondary antibodies (90 min, room temperature), and then detected with an ECL substrate followed by analysis in the Fusion software. Band analyses were performed in triplicate.

### 2.8 In vivo tumor models

Female BALB/c null mice (5 weeks old) were subcutaneously implanted in the right flank with 5×10^6^ stably transfected cells. The experiment was divided into three groups of eight mice each. Tumors were monitored for growth on a weekly basis, and tumors were obtained at 5 weeks after implantation when the mice were sacrificed by cervical dislocation under isoflurane anesthesia(The maximum size of the tumor was 2 mm^3^, The formula is V=(W^2^*L)2), weighed, and used for immunohistochemical staining, Western immunoblotting, and qPCR analyses.

Models of tumor metastasis were established by injecting stably transfected tumor cells into mice via the tail vein. At 7 weeks post-injection, liver imaging of mice was performed, followed by tumor dissection and the H&E staining of tissue sections.

### 2.9 Luciferase assays

Luciferase reporter plasmids were transfected into appropriate cells, after which a dual-luciferase reporter assay kit (Beyotime, RG027) was utilized for determination of luciferase activity. The luciferase plasmid structure is shown in the **Supplementary File**. Assays were conducted in triplicate.

### 2.10 Online database analysis

The database GEPIA2 (http://gepia2.cancer-pku.cn/#index) database was used for online data analysis, and the differential expression of genes and prognostic survival analysis chart were obtained.

### 2.11 RNA immunoprecipitation

Cells were harvested, lysed using RIP lysis Buffer for 30min at 4°C. Afterwards, the cell lysate was separated by centrifugation at 15000×g for 20 min for use. EPHB2 antibody was incubated with Protein A magnetic beads for 1h at 25°C, and then cell lysates were incubated with EPHB2 antibody and Protein A magnetic beads for 4 h at 25°C. This was followed by elution with Protein K at 55°C. The eluted complexes were then subjected to RNA extraction for subsequent quantification and analysis via qRT-PCR.

### 2.12 Data analyses

Results are reported as means ± SE and were compared in SPSS 26.0 (IBM, USA) using t-tests, ANOVAs, and Mann-Whitney U tests. Each independent experiment was performed three or more times. P < 0.05 was significant (*P < 0.05, **P < 0.01, ***P < 0.001).

## 3 Results

### 3.1 NPC exhibit the upregulation of tRF-1:28-Val-CAC-2, which promotes proliferative activity

**Figure 1:**
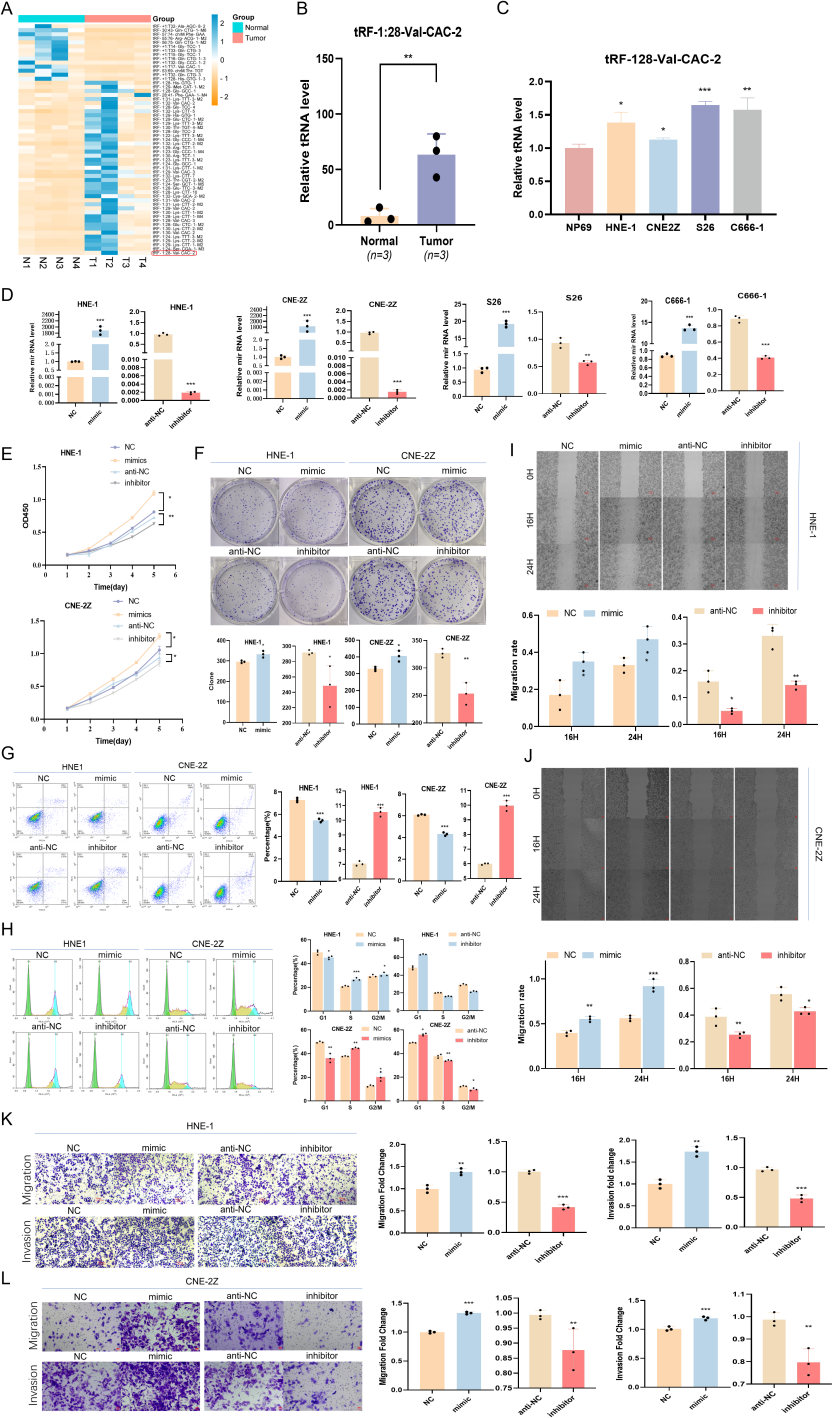
NPC exhibit the significant upregulation of tRF-1:28-Val-CAC-2, which promotes the development of NPC cell. **(A)** Expression heatmap of tRF (fold change of no less than 3 and p-value ≦̸ 0.05). **(B)** tRF-1:28-Val-CAC-2 was highly expressed in NPC tissues. **(C)** tRF expression in different NPC cells. **(D)** Gene transfection efficiency was measured by q-PCR. **(E-H)** The cell proliferation was detected by CCK-8 assays **(E)**, colony forming **(F)**, apoptosis **(G)** and cell cycle progression **(H)**. **(I-L)** Wound healing **(I, J)** and Transwell **(K, L)** assays were used to assess the relationship between tRF-1:28-Val-CAC-2 and NPC cell migration or invasivity. Data are mean ± SD, *P<0.05, **P<0.01, ***P<0.001.

In preliminary analyses (20) focused on the sequencing of tRF and tiRNA in NPC tumors and paracancerous tissues, tRF-1:28-Val-CAC-2 was identified as a novel tsRNA (**Figure 1A**), and its expression was found to be significantly elevated in NPC organizations (**Figure 1B**). Using RT-qPCR approach, significant increases in tRF-1:28-Val-CAC-2 expression at baseline were observed in various NPC cell lines (HNE1, CNE2Z, S26, C666-1) relative to control NP69 cells (**Figure 1C**), with the CNE2Z and HNE1 cells being selected for further experimental use. These two respective cell lines were then transfected with tRF-1:28-Val-CAC-2 inhibitor or mimic constructs to knock down or overexpress this tsRNA (**Figure 1D**). It was found by CCK-8 and colony formation assays that tRF-1:28-Val-CAC-2-mimic transfection enhanced NPC cell proliferation whereas its inhibition had the opposite effect (**Figures 1E, F**), supporting the importance of this tsRNA as a regulator of NPC cell proliferative growth.

### 3.2 tRF-1:28-Val-CAC-2 modulates NPC cell apoptosis and cell cycling

Through a flow cytometry-based approach, respective reductions and increases in levels of apoptotic death were observed in NPC cells transfected with tRF-1:28-Val-CAC-2 mimic and inhibitor constructs (**Figure 1G**). Moreover, the overexpression of this tsRNA enhanced the proportion of cells in the S-phase while decreasing the G1 phase frequency, whereas the opposite was observed following its inhibition, thus suggesting that tRF-1:28-Val-CAC-2 serves as a key regulator of survival and cell cycle progression (**Figure 1H**).

### 3.3 tRF-1:28-Val-CAC-2 regulates invasive and migratory activity in NPC cells

In wound healing assays (**Figure 1I**), tRF-1:28-Val-CAC-2 mimic and inhibitor transfection respectively enhanced and suppressed NPC cell migration after 24 h. Consistently, the overexpression of this tsRNA led to enhanced migration and invasivity in the Transwell assay while its knockdown impaired such activity, emphasizing the important role that tRF-1:28-Val-CAC-2 plays as a regulator of the malignant growth of these tumor cells (**Figure 1J**).

### 3.4 tRF-1:28-Val-CAC-2 regulates EMT activity in NPC cells

**Figure 2:**
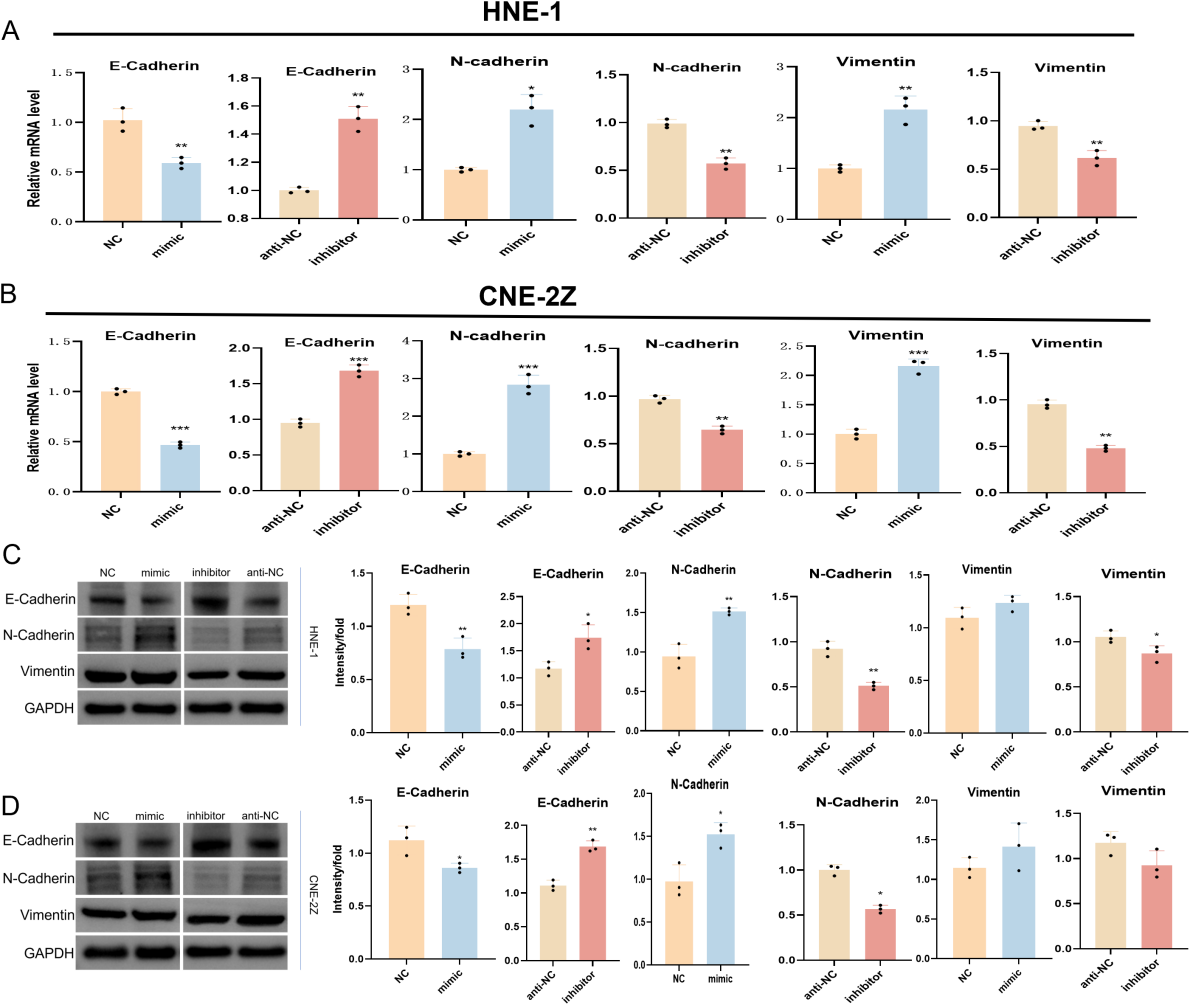
Effect of tRF-1:28-Val-CAC-2 on EMT. The impact of tRF-1:28-Val-CAC-2 levels on EMT-related marker expression was assessed via qPCR **(A, B)** and Western immunoblotting **(C, D)**. Data are mean ± SD, *P<0.05, **P<0.01, ***P<0.001.

Next, the expression of EMT-associated markers within NPC cells was evaluated through Western immunoblotting and qPCR approaches. In cells transfected with the tRF-1:28-Val-CAC-2 mimic, both mRNA and protein expression of E-cadherin was reduced, whereas Vimentin and N-cadherin levels were increased. The converse phenotypes were evident following the inhibition of this tsRNA, indicating that tRF-1:28-Val-CAC-2 can promote EMT induction in NPC cells (**Figure 2**).

### 3.5 tRF-1:28-Val-CAC-2 supports more robust xenograft tumor growth in mice

**Figure 3:**
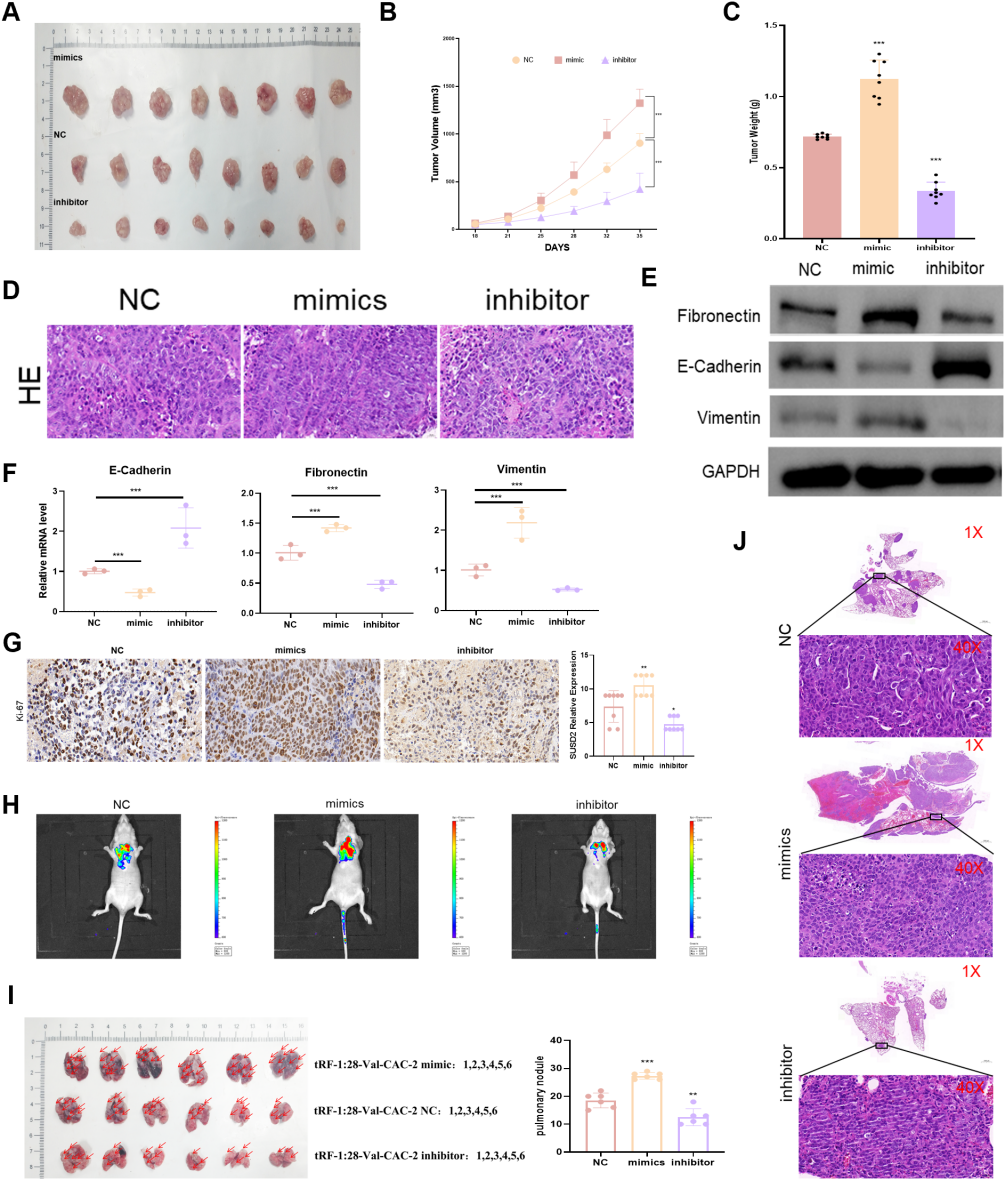
tRF-1:28-Val-CAC-2 regulates NPC tumor growth *in vivo*. **(A)** Subcutaneous tumor growth was analyzed in nude mice. **(B, C)** The impact of tRF-1:28-Val-CAC-2 on tumor volume and weight following subcutaneous implantation was quantified. **(D)** H&E staining of subcutaneous tumors. **(E, F)** Intratumoral EMT-related marker expression was assessed via Western immunoblotting **(E)** and qPCR **(F)**. **(G)** Immunohistochemistry was used to detect Ki-67. **(H)** Bioluminescence *in vivo* imaging of mouse lung tissue. **(I)** Images of Lung metastatic tumor tissue in nude mice. **(J)** H&E stained lung metastases. Data are means ± SD, *P<0.05, **P<0.01, ***P<0.001.

To gain further confirmation of the ability of tRF-1:28-Val-CAC-2 to regulate NPC cell malignancy, animal model studies were next conducted. In the tRF-1:28-Val-CAC-2 mimic group, a significant increase in tumor weight was evident relative to control mice, whereas the weight of tumors expressing the tRF-1:28-Val-CAC-2 inhibitor construct was significantly reduced (**Figures 3A–D**). Corresponding significant reductions and increases in E-cadherin expression were evident in the tRF-1:28-Val-CAC-2 mimic and inhibitor groups relative to control cells, while the opposite pattern was observed for vimentin and fibronectin expression (**Figures 3E, F**). Significantly increased intratumoral Ki67 expression was evident in tumors from the tRF-1:28-Val-CAC-2 mimic group relative to control tumors, while this activity was reduced following tRF-1:28-Val-CAC-2 inhibition (**Figure 3G**). In a model of lung metastasis, the tRF-1:28-Val-CAC-2 mimic group presented with significant increases in tumor weight and nodule size, whereas these were significantly reduced relative to control mice in the tRF-1:28-Val-CAC-2 inhibitor group (**Figures 3H–J**).

### 3.6 tRF-1:28-Val-CAC-2 interacts with EPHB2

**Figure 4:**
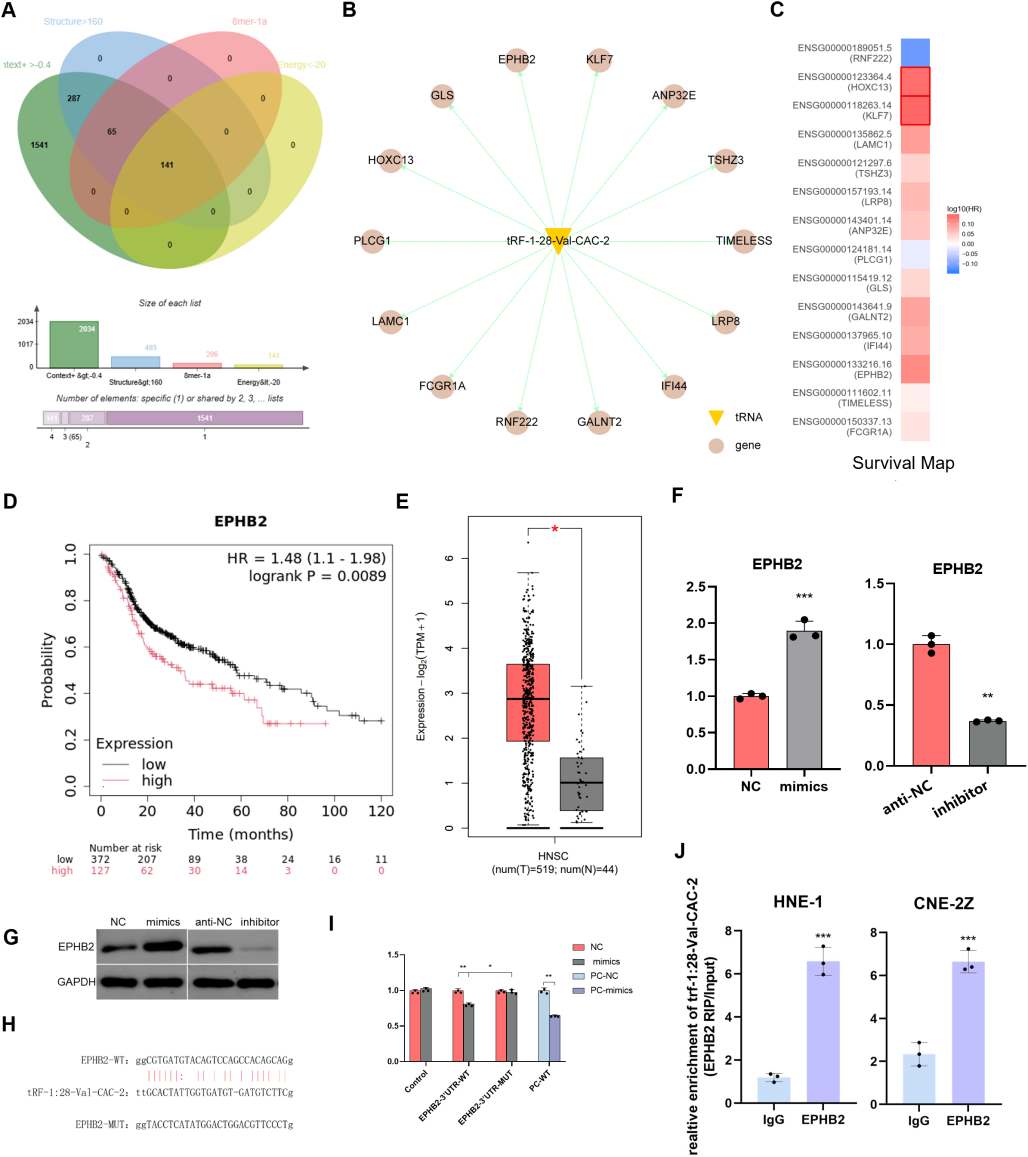
EPHB2 is highly expressed in NPC and targets to tRF-1:28-Val-CAC-2. **(A)** The predicted target genes were further screened. **(B)** Fourteen potential target genes were predicted. **(C)** GEPIA 2 online database analyzes survival map. **(D)** Prognostic survival curve of EPHB2 **(E)** GEPIA 2 online database analyzes EPHB2 expression in NPC. **(F, G)** Effect of tRF on EPHB2 expression was detected via RT-qPCR **(F)** and Western immunoblotting **(G)**. **(H)** Binding site map. **(I)** Luciferase reporter assays. **(J)** RNA immunoprecipitation assays. Data are means ± SD, *P<0.05, **P<0.01, ***P<0.001.

In the preliminary stage of the study (20), a total of 2093 genes were screened using the TargetScan tool. Furthermore, 14 potential target genes were identified according to the following criteria: Context+>-0.4, structure>160, 8 mer-1a≥1, Energy<-20 (**Figures 4A, B**). The expressions of these genes were all found to be associated with prognosis (**Figure 4C**). Further investigation revealed that EPHB2 exhibited elevated expression in head and neck tumors, which was associated with a poorer prognosis (**Figures 4D, E**). RT-qPCR and western immunoblotting experiments demonstrated that the level of tRF-1:28-Val-CAC-2 could significantly affect the expression of EPHB2 (**Figures 4F, G**). To this end, we performed binding site prediction, dual luciferase reporter gene assays and RNA immunoprecipitation assays to validate the binding (**Figures 4H–J**).

### 3.7 tRF-1:28-Val-CAC-2 targets EPHB2 to control NPC cell phenotypes

**Figure 5:**
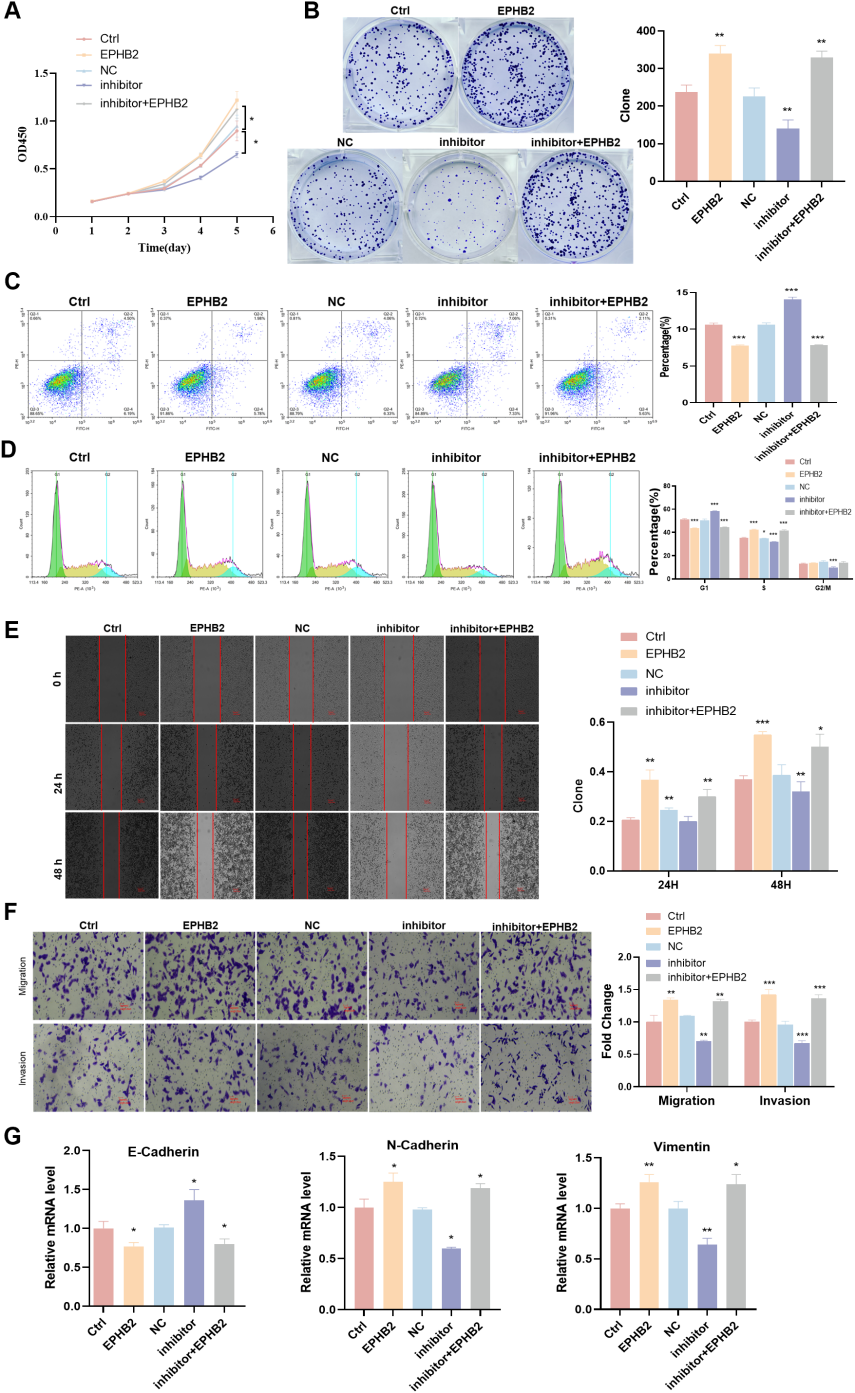
tRF-1:28-Val-CAC-2 binds to EPHB2 to modulate NPC cell behavior. **(A, B)** CCK-8 **(A)** and colony formation **(B)** assays for cellular proliferation. **(C, D)** Flow cytometry assessing apoptosis **(C)** and cell cycle progression **(D)**. **(E, F)** Migration and invasion indicated by wound healing **(E)** and Transwell **(F)** assays. **(G)** RT-qPCR analysis of EMT-related gene expression. Data are means ± SD, *P<0.05, **P<0.01, ***P<0.001.

To better understand how tRF-1:28-Val-CAC-2 and EPHB2 are related to one another, cell-level rescue experiments were next performed. In co-transfection experiments using in which tRF-1:28-Val-CAC-2 inhibitor and EPHB2 constructs were delivered to cells, overexpression EPHB2 was found to significantly enhance HNE1 cell proliferative, apoptosis, migratory, and invasive growth (**Figures 5A–F**), this is the exact opposite of the inhibitory effect shown in the tRF-1:28-Val-CAC-2 inhibitor group. The mRNA level of E-cadherin was significantly reduced in the siEPHB2 and tRF-1:28-Val-CAC-2 inhibitor + EPHB2 groups relative to Ctrl group, while the opposite trends were observed for N-cadherin and Vimentin expression (**Figure 5G**).

**Figure 6:**
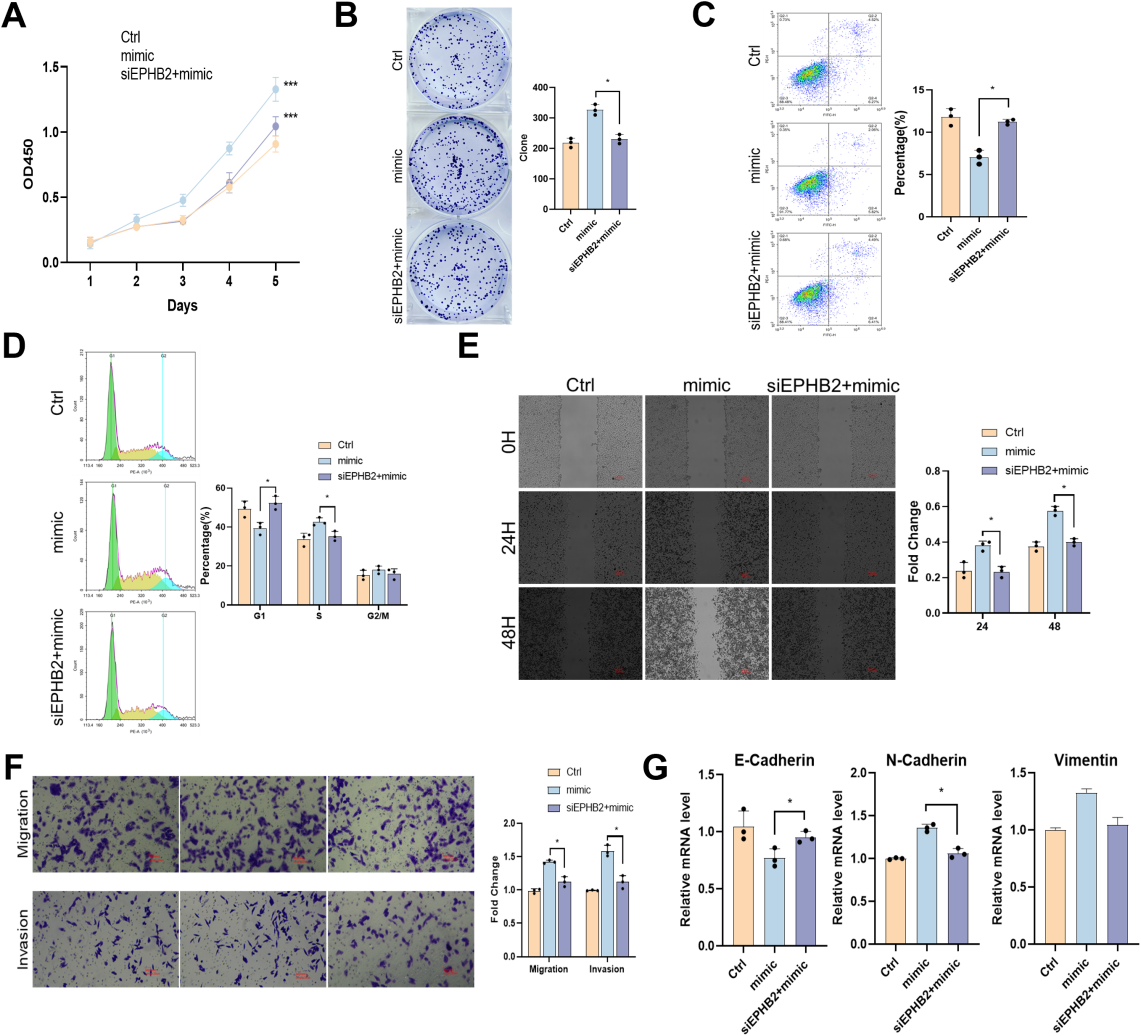
tRF-1:28-Val-CAC-2 binds to EPHB2 to modulate NPC cell behavior. **(A, B)** CCK-8 **(A)** and colony formation **(B)** assays for cellular proliferation. **(C, D)** Flow cytometry assessing apoptosis **(C)** and cell cycle progression **(D)**. **(E, F)** Migration and invasion indicated by wound healing **(E)** and Transwell **(F)** assays. **(G)** RT-qPCR analysis of EMT-related gene expression. Data are means ± SD, *P<0.05, **P<0.01, ***P<0.001.

We knocked down EPHB2 gene in nasopharyngeal carcinoma cell HNE1 that overexpressed tRF-1:28-Val-CAC 2, and the results were similar to those described above. After EPHB2 gene was knocked down, the promotion of proliferation, migration and invasion of tRF-1:28-Val-CAC-2 was inhibited (**Figure 6**). Therefore, EPHB2 may be the downstream target gene of tRF-1:28-Val-CAC-2.

## 4 Discussion

NPC remains one of the most prevalent cancers of the neck and head, with squamous cell carcinoma cases comprising over 95% of these diagnoses. Despite extensive research focused on these tumors, efforts to prevent NPC metastasis and recurrence still face major clinical challenges, emphasizing the limited nature of current understanding regarding the mechanistic basis for disease progression. Despite the value of pathological examination and imaging in the context of diagnosis and staging, their utility when seeking to guide the personalized treatment of individuals with NPC remains fairly restricted (1). There is thus a clear need to define objective biomarkers that offer greater diagnostic and prognostic utility. Our team performed high-throughput sequencing analyses that led to the identification of tRF-1:28-Val-CAC-2 as a novel tsRNA capable of significantly promoting NPC cell malignancy. Target gene prediction efforts uncovered the ability of the EPHB2 3’-UTR to be directly targeted by tRF-1:28-Val-CAC-2. The overexpression of EPHB2 mediated by tRF-1:28-Val-CAC-2 in NPC cells was ultimately found to drive their malignant growth, providing a promising new avenue for efforts to diagnose and treat this deadly disease.

Increasingly advanced sequencing technologies and workflows have led to the recognition that a large number of non-coding RNAs are important regulators of cancer in humans. Over 50% of tRFs and tsRNAs are now thought to serve as key mediators of tumorigenesis such that they have been proposed as valuable targets for diagnostic assessment or therapeutic intervention (21). However, the link between tRFs and NPC remains poorly researched. In preliminary analyses, our team identified three tRFs that were differentially expressed in NPC, including tRF-1:28-Val-CAC-2 and tRF-1:24-Ser-CGA-1-M3, which were upregulated, as well as tRF-55:76-Arg-ACG-1-M2, which was downregulated. ROC analyses revealed that tRF-1:28-Val-CAC-2 yielded a high degree of diagnostic accuracy when detecting cases of primary NPC, providing an AUC of 0.732 (95% CI 0.599-0.865), a sensitivity of 80%, and a specificity of 70%. This suggests that tRF-1:28-Val-CAC-2 may be a diagnostic marker for NPC. *In vitro* assays demonstrated that tRF-1:28-Val-CAC-2 serves as a key regulator of tumorigenic behavior in NPC cells, it promotes the proliferation, migration and invasion of nasopharyngeal carcinoma cells. This tsRNA was also found to suppress apoptotic NPC cell death and to alter EMT-related gene expression when overexpressed in NPC cells. *In vivo*, tRF-1:28-Val-CAC-2 was also able to promote enhanced NPC tumor growth.

Based on these results, an effort was made to clarify the specific mechanisms through which tRF-1:28-Val-CAC-2 favors NPC malignancy NPC. Predictive analyses revealed several putative targets with the potential to interact with tRF-1:28-Val-CAC-2, EPHB2 is one of the potential targets, and high expression of EPHB2 predicts poor clinical outcomes in NPC patients. Meanwhile, qPCR and western blotting showed that the expression of EPHB2 is different between tRF-1:28-Val-CAC-2 inhibited and overexpressed NPC cells (**Figure 4**), and this tsRNA was ultimately confirmed to interact with EPHB2 in a dual-luciferase reporter assay. Through rescue experiments, a functional relationship between tRF-1:28-Val-CAC-2 and EPHB2 was confirmed, The expression of EMT-related genes was detected in EPHB2 overexpressing NPC cells, tRF-1:28-Val-CAC-2 inhibited cells, and both tRF-1:28-Val-CAC-2 inhibited and EPHB2 overexpressing cells suggesting that the interaction between the two influences NPC-related EMT induction and proliferative tumor growth.

EPHB2 is an EPH receptor family member that regulates a range of cellular processes including adhesion, shape determination, migration, and cytoskeletal dynamics (22). EPH receptors are increasingly believed to serve as key regulators of tumorigenesis such that they hold promise as targets for the study of tumor-related biomarkers and therapeutic resistance (23–26). EPHB2 has been reported to promote migratory and invasive activity in breast (27) and cervical cancers (28), whereas its effects are instead inhibitory in colorectal (29), lung adenocarcinoma (30) and bladder cancers (31). In this study, the tRF-1:28-Val-CAC-2-mediated overexpression of EPHB2 was associated with EMT induction and enhanced NPC cell proliferation.

In conclusion, these results demonstrate that tRF-1:28-Val-CAC-2 is capable of promoting proliferation and EMT induction in NPC cells through its ability to target and suppress (**Figure 6**). It can additionally suppress the apoptotic death of cancer cells. Given these findings, tRF-1:28-Val-CAC-2 holds promise as a novel target for efforts to diagnose and/or treat NPC, However, how to apply it to clinical practice is still the focus of research, Whether precise delivery of tumor suppressor tsRNAs or targeting tRF-1:28-Val-CAC-2 expression with drugs will require further investigation and rigorous preclinical testing. Therefore, tRF-1:28-Val-CAC-2 still has great potential in the clinical diagnosis and treatment of NPC.

## Data Availability

The datasets presented in this study can be found in online repositories. The names of the repository/repositories and accession number(s) can be found below: https://www.ncbi.nlm.nih.gov/geo/query/acc.cgi?acc=GSE159746.
