# Correction: tRF-1:28-Val-CAC-2 promotes the development of nasopharyngeal cancer by targeting EPHB2

**DOI:** 10.3389/fonc.2025.1672669

**Published:** 2025-08-13

**Authors:** Hui Li, Xiaomin Wang, Anchi Sun, Weiwei Liu, Rongrong Lv, Mingjie Zhang, Zhiwei Xing, Shiyin Ma, Yehai Liu, Kai Zhang

**Affiliations:** ^1^ Department of Otolaryngology-Head and Neck Surgery, The First Affiliated Hospital of Anhui Medical University, Hefei, Anhui, China; ^2^ Department of Otolaryngology-Head and Neck Surgery, The First Affiliated Hospital of Bengbu Medical University, Bengbu, Anhui, China; ^3^ Anhui Engineering Technology Research Center of Biochemical Pharmaceutical, Bengbu, Anhui, China; ^4^ Department of Stomatology, The First Affiliated Hospital of Bengbu Medical University, Bengbu, Anhui, China

**Keywords:** tRF-1:28-Val-CAC-2, *EPHB2*, NPC, tsRNA, EMT

In the published article, there was an error in affiliation 1. Instead of “Anhui Medical University, Hefei, Anhui, China”, it should be “Department of Otolaryngology-Head and Neck Surgery, The First Affiliated Hospital of Anhui Medical University, Hefei, Anhui, China”.

The original version of this article has been updated.

