# Supplementary material for: tRF-1:28-Val-CAC-2 promotes the development of nasopharyngeal cancer by targeting EPHB2
Source: Front Oncol. 2025 May 23;15:1564601. doi: 10.3389/fonc.2025.1564601 (PMC12141329; doi:10.3389/fonc.2025.1564601)
Supplement: Supplementary file 2 [file Supplementaryfile2.doc]

Report on the construction experiment of gene plasmids

## I. Experimental Purpose

## The regulation of the reporter gene by the stimulating factor is determined by detecting the luciferase activity.

## II. Experimental Principle

## Taking advantage of the characteristic that luciferase combines with substrates to undergo chemiluminescence reactions, the regulatory element was cloned downstream of the sea kidney luciferase gene to construct a luciferase reporter plasmid. The influence of the experiment on the regulatory elements was determined by measuring the luciferase activity.

## III. Experimental Materials

1．Gene information

gene name：EPHB2

2. Carrier information

Name：psicheck2.0

Component order：SV40-RLuc-MCS-pHSVTK-fLuc

Prokaryotic resistance：Amp


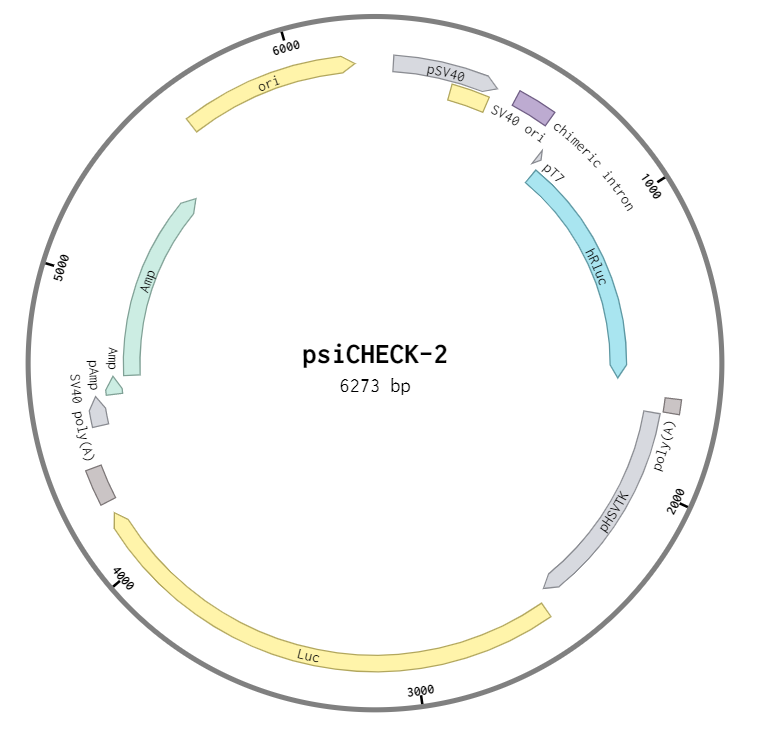
Vector mapping：

3． Main reagents and consumables

| **Name of the reagent consumable** | **Brand or producer** |
| --- | --- |
| Plasmid mini-pick kit | OMEGA |
| Plasmid mini-pick kit | TransGen Biotech |

## IV、Experimental Procedure

1．Design the cloning site

Design the cloning site：XhoⅠ（CTCGAG）

3' digestion site：NotⅠ（GCGGCCGC）

2．Obtain the Destination Sequence

NCBI：EPHB2（GENE ID:2048）

3. Target prediction

According to the tRF-1:28-Val-CAC-2 sequence provided by the customer, the target was predictively analyzed.

Mutation site design：

binging site：GGCGTGATGTACAGTCCAGCCACAGCAGG

mut： GGTACCTCATATGGACTGGACGTTCCCTG

4．Objective sequence synthesis

According to the truncated sequence of the EPHB2 gene, the sequence was synthesized by General Biology.

5. 亚克隆

According to the designed digestion site, the target sequence was subcloned into the vector psicheck 2.0, and the specific report is shown in the table below.

| **Certificate of Analysis** | | | | | | | | | |
| --- | --- | --- | --- | --- | --- | --- | --- | --- | --- |
| **Gene Name** | EPHB2-mut | | | **Cloning Vector** | | | pSiCheck2 .0 | | |
| **Cloning Sites** | XhoI(CTCGAG)-NotI(GCGGCCGC) | | | **Insert Size** | | | 360bp | | |
| **Competence** | Top10 | | | **Vector Resistance** | | | Amp | | |
| **QC Results** | | | | | | | | | |
| **Test Items** | | **Specifications** | | | | | | | **Results** |
| **Insert Sequence** | | Insert sequence results consistent with target | | | | | | | Pass |
| **Vector Sequence** | | Flanking sequence consistent with expected | | | | | | | Pass |
| **ORF Across Junction** | | Correct and consistent with target | | | | | | | N/A |
| **Restriction Digest** | | Expected fragment sizes observed | | | | | | | Pass |
| **PCR Amplification** | | Correct without non - specific bands | | | | | | | N/A |
| **DNA Quantity/Quality** | | Actual yield (by A 260 ) | | | | | | | 5ug |
|  |  | Concentration (n/a if lyophilized) | | | | | | | N/A |
|  |  | Purity (A 260/A280 =1.8 - 2.0) | | | | | | | Pass |
|  |  | # of Tubes | | | | | | | 1 |
|  |  | Matrix | | | | | | | TE (lyophilized) |
| **Endotoxin Test** | | Verified, <0.1 EU/µg (Endo-Free Preps Only) | | | | | | | N/A |
| **Appearance** | | Clear, no visible particles | | | | | | | Pass |
| **Label** | | Correct and white | | | | | | | Pass |
| **Comments** | | - | | | | | | | - |
| **Restriction Digestion Map** | | | | | | | | | |
|  | | | **1** | | **2** | **M** | |  | |
| 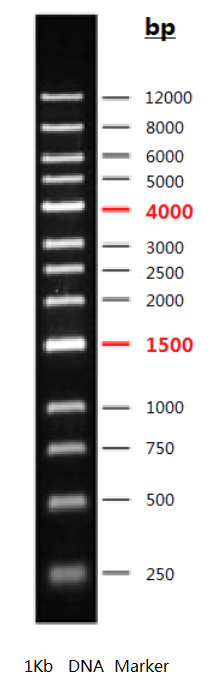 | | | 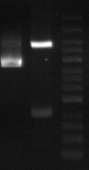 | | | | | **Lane 1：**Plasmid  **Lane 2**: Plasmid Digested with XhoI-EcoRI  **Lane M**: DNA Marker | |

6. Recombinant plasmid amplification

1) Pick the correct recombinant plasmid for general sequencing monoclonal colony expansion culture;

2) Transfer the bacterial solution to 5ml LB medium at 37°C, 220rpm overnight;

3) Plasmid extraction was performed using the OMEGA endotoxin-free plasmid mini-extract kit;

4) Determine plasmid A260/280 with concentration and store at -20 °C.

## 五、测序结果

AGCTTCGTGGAGCGCGTGCTGAAGAACGAGCAGTAATTCTAGGCGATCGctcgagGCTGTCAGGGAGGGCTTCATGGAGAGGCTGTCTTGAAAATGACAGAGGTCAAAAGAAAGGTCACTCCAAGACGCAGCTGCCAGAATGGTCCAACACAGTGAGGAGTTGTGTCTGGATGGGCCAGTGGAACGGGGGAAGTGAAGGTTGATATAAGTGGAGGTTGAGGCAGTTTAGGTACCTCATATGGACTGGACGTTCCCTGTTCTTGAGCAGGAGGGTAGCATAGTGAGCATCAGGTTCTAGGAAGAAGCACCAGTTCAGCCATCAGATGGGGCAGGATGCCTCCCAGCTACTCCTCTCCCCGAGAAGGAATTGCCCCCGGAGCGGCCCTCATTTATTCCAGGAGgcggccgcTGGCCGCAATAAAATATCTTTATTTTCATTACATCTGTGTGTTGGTTTTTTGTGTGAGGATCTAAATGAGTCTTCGGACCTCGCGGGGGCCGCTTAAGCGGTGGTTAGGGTTTGTCTGACGCGGGGG
